# Management of mild traumatic brain injury–trauma energy level and medical history as possible predictors for intracranial hemorrhage

**DOI:** 10.1007/s00068-018-0941-8

**Published:** 2018-03-17

**Authors:** Tomas Vedin, Sebastian Svensson, Marcus Edelhamre, Mathias Karlsson, Mikael Bergenheim, Per-Anders Larsson

**Affiliations:** 1 Clinical Sciences, Helsingborg, Lunds Universitet, Svartbrödragränden 3-5, 251 87 Helsingborg, Sweden; 2 Centralsjukshuset i Karlstad, Karolinska Institute, Rosenborgsgatan 9, 652 30 Karlstad, Sweden; 3 Centralsjukshuset i Karlstad, Umeå University, Rosenborgsgatan 9, 652 30 Karlstad, Sweden

**Keywords:** Brain injuries, Traumatic, Epidemiology, Practice guidelines as topic, Intracranial hemorrhage, Traumatic, S100B Calcium Binding Protein Beta Subunit

## Abstract

**Purpose:**

Head trauma is common in the emergency department. Identifying the few patients with serious injuries is time consuming and leads to many computerized tomographies (CTs). Reducing the number of CTs would reduce cost and radiation. The aim of this study was to evaluate the characteristics of adults with head trauma over a 1-year period to identify clinical features predicting intracranial hemorrhage.

**Methods:**

Medical record data have been collected retrospectively in adult patients with traumatic brain injury. A total of 1638 patients over a period of 384 days were reviewed, and 33 parameters were extracted. Patients with high-energy multitrauma managed with ATLS™ were excluded. The analysis was done with emphasis on patient history, clinical findings, and epidemiological traits. Logistic regression and descriptive statistics were applied.

**Results:**

Median age was 58 years (18–101, IQR 35–77). High age, minor head injury, new neurological deficits, and low trauma energy level correlated with intracranial hemorrhage. Patients younger than 59 years, without anticoagulation or antiplatelet therapy who suffered low-energy trauma, had no intracranial hemorrhages. The hemorrhage frequency in the entire cohort was 4.3% (70/1638). In subgroup taking anticoagulants, the frequency of intracranial hemorrhage was 8.6% (10/116), and in the platelet-inhibitor subgroup, it was 11.8% (20/169).

**Conclusion:**

This study demonstrates that patients younger than 59 years with low-energy head trauma, who were not on anticoagulants or platelet inhibitors could possibly be discharged based on patient history. Maybe, there is no need for as extensive medical examination as currently recommended. These findings merit further studies.

## Introduction

During the twentieth century, patients with traumatic brain injury and loss of consciousness presenting in the emergency department were admitted for further observation. If they deteriorated during observation, computerized tomography (CT) was performed. Modern guidelines for managing traumatic brain injury in the emergency room have been utilized now for well over a decade and has alleviated clinical practice. When these guidelines are applied correctly, approximately 50% of the patients undergo head CTs [1].

Repetitive CT of the head may be related to adverse effects later in life. Even though modern CT machines are using less and less radiation, X-ray-induced cancer is something the clinician must take into account [2–5].

Modern guidelines are based on the previous epidemiological studies, which have shown a bimodal distribution of age, with the lowest incidence around 55 years of age. The highest incidence in the adult population is seen among young adults (predominantly males) and the elderly [6–12].

A randomized, controlled study from the same era showed that upfront CT when presenting in the emergency department was sufficient to rule out intracranial hemorrhage in most patients. They could be safely discharged [13]. North American studies have shown corresponding results, which have led to the establishment of the Canadian CT Head Rule and New Orleans Criteria [1, 14]. In the previous studies, the reported frequency of intracranial hemorrhage was 8% [1, 15], Mortality ranged from 0.1 to 0.7% [6, 15].

The common denominator for all degrees of traumatic brain injury is that the patient has sustained mechanical energy to the head from external physical force. However, trauma energy level varies. Both academic definitions and clinical guidelines are based primarily on level of consciousness, degree of head injury, and the temporal progression of level of consciousness. Neither the Canadian CT Head Rule, the New Orleans Criteria, nor the Scandinavian Neurotrauma Committee guidelines base decisions to dismiss patients on patient-stated trauma energy alone, suggesting that clinical status is the most important factor [1]. These guidelines use clinical features mostly as “rule-in criteria” for intracranial hemorrhage, giving patient history a subordinate role in relation with clinical status.

If patient history alone could be used as a “rule-out criterion” for intracranial hemorrhage, emergency department workloads and radiation levels could be reduced, resulting in both economic and public health benefits.

The aim of this study was to evaluate the characteristics of adults presenting with the chief complaint of head trauma over a 1-year period to identify clinical features predicting intracranial hemorrhage. The analysis was done with particular emphasis on patient history, clinical findings, and epidemiological traits.

## Methods

The study was conducted as a retrospective analysis of medical records of patients presenting with head trauma at the emergency department of Helsingborg General Hospital. This hospital serves a geographic area of 350,000 people. The emergency department has approximately 72,000 visits annually. It has trauma surgeons, general surgeons, orthopedic surgeons, emergency medicine doctors, anesthesiologists, and otorhinolaryngologists. The nearest neurosurgical clinic is 40 km away. Multitrauma patients are managed according to ATLS™.

When presenting to the emergency department, patients were registered in an electronic patient registry. Its purpose was to keep track of room location and chief complaint. After extracting a list of all patients 18 years or older who registered with the chief complaint “head injury,” we reviewed the medical records. Patients may have had other minor injuries as well, but were not initially classified as multitrauma. High-energy multitrauma patients (*n* = 647) were excluded to make the patient cohort more representative of the majority of emergency-room patients with head injury. Furthermore, the hospital manages multitrauma patients with a different algorithm, which we did not seek to evaluate. The review was performed on patients registered between November 11, 2013, and November 30, 2014, a total of 384 days.

The following parameters were manually extracted from medical records:

1. Age (years).
2. Gender (m/f).
3. Head CT performed (yes/no).
4. Head CT outcome (hemorrhage/no hemorrhage).
5. Admission to general hospital ward (yes/no).
6. Admission to intensive care unit (ICU) (yes/no).
7. Admission to neuro intensive care unit (neuro ICU) (yes/no).
8. Neurosurgical intervention (yes/no).
9. Degree of head injury (minimal, mild, moderate, and severe).
10. Level of consciousness using Reaction Level Scale 85 (RLS85) (1–8).
11. Level of consciousness using Glasgow Coma Scale (GCS) (15–3).
12. Blood pressure (systolic mm Hg/diastolic mm Hg).
13. Pulse rate (beats/minute).
14. Size of pupils (mm).
15. Body weight (kilograms).
16. Height (meters).
17. Past medical history (yes/no).
18. Anticoagulant treatment (no/warfarin/noac/injection).
19. Platelet inhibitor treatment (no/aspirin/clopidogrel/ticagrelor/other).
20. Other medication (yes/no).
21. Preexisting/new focal neurological deficits (yes/no).
22. Deterioration of neurological status during observation (yes/no).
23. S100B level (µg/L).
24. Nausea (yes/no).
25. Vomiting (yes/no).
26. Number of vomits (n).
27. Amnesia, type and duration (yes/no, antegrade/retrograde, time hh:mm).
28. Loss of consciousness (yes/no).
29. Peritraumatic seizure (yes/no).
30. Posttraumatic headache (yes/no).
31. Increasing intensity of headache (yes/no).
32. Trauma energy levels (low, medium, and high).
33. Clinical signs of basal skull fracture (yes/no).
34. Orthostatic hypotension (yes/no).
35. Cardiac dysrhythmia (yes/no).
36. Time from injury to medical examination at the emergency department (hh:mm).
37. Influence of any or multiple drugs/alcohol (yes/no).

Level of trauma energy (see #32 above) was interpreted on the basis of trauma mechanism, as follows:

- Low [1]: fall from less than 1 m or fewer than 5 stairs.
- Medium [1]: fall from 1 to 3 m or 5 or more stairs.
- High (ATLS^™^): fall from 3 m or more, motor vehicle accident at 70 km/h or more with seatbelt, motor vehicle accident at 30 km/h or more without seatbelt, any motorcycle accident, pedestrian hit by motor vehicle.

Minimal traumatic brain injury is trauma to the head without loss of consciousness and without any of the following criteria: amnesia, nausea, vomiting, vertigo, or focal neurological deficits [16].

Mild traumatic brain injury is an acute brain injury resulting from mechanical energy to the head from external physical forces. Operational criteria for clinical identification consist of the following:

1. One or more of the following: confusion or disorientation, loss of consciousness for 30 min or less, posttraumatic amnesia for less than 24 h, and other transient neurological abnormalities such as focal signs, seizure, and intracranial lesion not requiring surgery
2. GCS score of 13–15 either 30 min after injury or later upon presentation for healthcare [8].

Moderate traumatic brain injury is defined as a brain injury resulting in a loss of consciousness from 20 min to 6 h and a GCS score of 9–12 [17].

Severe traumatic brain injury is defined as a brain injury resulting in loss of consciousness for more than 6 h and a GCS score of 3–8 [17].

### Statistics

Data were analyzed using SPSS version 21 for Mac. Q–Q plots and the Shapiro–Wilk formula were used to test for normal distribution. Central tendencies are presented as medians when skewed. Post hoc multivariate analysis was performed on all relevant and applicable parameters; this analysis was performed via univariate binomial logistic regression and the insertion of significant parameters (*p* < 0.4) in a multivariate logistic regression model. Another multivariate logistic regression was performed on the significant parameters (*p* < 0.05) of the first multivariate regression. Missing data were replaced by series median. Post hoc subgroup analysis with descriptive statistics was performed to ascertain the effect of different parameters on intracranial hemorrhage to determine the clinically significant age cutoff.

## Results

The inclusion criteria were satisfied by 1,638 patients. Of the 1,638 patients, 456 (27.8%) had minimal head injury, 922 (56.3%) had mild head injury, 18 (1.1%) had moderate head injury, 4 (0.2%) had severe head injury, and 238 (14.5%) were not assessable. Twelve patients were admitted to intensive care unit and 5 of these had neurosurgical intervention. Level of consciousness was classified according to the Scandinavian reaction level scale (RLS-85) in 1608 (98.2%) cases. Only 47 cases (2.7%) were classified according to GCS.

**Fig. 1:**
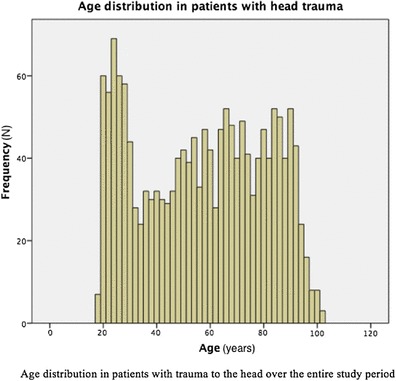
Age distribution in patients with head trauma

During the study period, 842 CTs were performed, representing 51.4% of the patients. Of these, 588 (69.8%) were performed on patients with low-energy trauma. The median age of patients who did not undergo CT was 48 years (18–101, IQR 28–67) and that of patients who underwent CT was 67 years (18–100, IQR 47–83). See Fig. 1 for age distribution of entire cohort.

In multivariate logistic regression of 12 applicable predictor variables, only 4 were statistically significant: high age, minor head injury, new neurological deficits, and low trauma energy level (Table 1).

**Table 1:** Regression analysis of potential parameters predicting intracranial hemorrhage

Parameter	p-value		
	Univariate regression	Multivariate regression 1	Multivariate Regression 2
Age	< **0.001**	< **0.001**	< **0.001**
Gender	**0.094**	**0.003**	0.091
Degree of head injury	< **0.001**	< **0.001**	< **0.001**
Level of consciousness (RLS)	**0.016**	0.469	n/a**
Past illness	**0.015**	0.792	n/a**
Anticoagulation treatment	**0.013**	0.214	n/a**
Platelet inhibitor	< **0.001**	0.111	n/a**
Current medication	0.616	n/a*	n/a**
Preexisting neurological deficits	0.957	n/a*	n/a**
New neurological deficits	< **0.001**	**0.007**	< **0.001**
Trauma energy level	< **0.001**	< **0.001**	< **0.001**
Intoxication	**0.034**	0.194	n/a**

Bold values indicate statistically significant

*Value is marked n/a when significance lever in the previous analysis was too low for inclusion in the next statistical analysis (*p* < 0.4)

**Value is marked n/a when significance lever in the previous analysis was too low for inclusion in the next statistical analysis (*p* < 0.05)

All patients with moderate or severe head injury underwent CT scan. Some parameters were excluded from analysis, because they occurred after head CT was performed or were not relevant to analysis of predictors for intracranial hemorrhage (admission to intensive care unit/neurointensive care unit/general ward, time at emergency room, time until admitted, and neurosurgical intervention). Some parameters were excluded due to > 30% missing values [level of consciousness (GCS), blood pressure (systolic and diastolic), pulse rate, weight, height, size of pupils, s100b level, loss of consciousness, nausea, vomiting, number of vomits, amnesia type and duration, seizures, headache on admission, worsening headache, orthostatic hypotension, cardiac dysrhythmia, time from trauma to medical examination and signs of basal skull fracture].

In the 58 years or younger subgroup (*n* = 826/1638 [50.4%]), no intracranial hemorrhage was found in patients with low-energy trauma. When not stratifying this age cohort according to intracranial hemorrhage, patients with all degrees of head injury were found. No patients in this subgroup had anticoagulants or platelet inhibitors. Of the patients with intracranial hemorrhage, two were missing the trauma-energy parameter (Table 2).

**Table 2:** Age, energy level, and frequency of intracranial hemorrhage

	Age (years)*				*N* Total
	18–39	40–58	59–79	80–101	
	*N* Intracranial hemorrhage/*N* total cohort				
Energy level					
Low	0/328	0/251	16/349	27/323	1251
Medium	0/47	3/28	5/17	2/2	94
High	2/11	1/3	0/0	2/2	16
n/a	1/93	1/65	5/75	5/44	277
*N* patients with intracranial hemorrhage					
	3/479 (0.6%)	5/347 (1.4%)	26/441 (5.9%)	36/371 (9.7%)	70

*Group cutoffs were selected at middle age (40 years), first occurrence of intracranial hemorrhage in low-energy trauma group (59 years), and age when intracranial hemorrhages are much more common (80 years)

Further analysis of these two patients revealed that both had sustained the head injury while intoxicated and were unable to report the trauma mechanism. One of these patients, from the 18–39 age group, was injured during a fist fight, and the other, from the 40–58 age group, was injured in unclear circumstances. Both had ongoing intravenous substance abuse, and both presented to the emergency room several days after trauma.

353 patients (21.6%) were admitted to the surgical ward due to head trauma. Seven (0.4%) patients were cared for at the ICU and five (0.3%) patients underwent neurosurgical intervention and received care at the neuro ICU. 70/1638 (4.3%) patients had intracranial hemorrhage. Of the patients undergoing CT scan, 8.3% had intracranial hemorrhage. 76% of patients sustained low-energy force. 6% sustained medium energy trauma, 1.5% sustained high-energy trauma, and in 17.5% of cases, it was not assessable.

The distribution of intracranial hemorrhage was 45/70 (64.3%) in males and 25/70 (35.7%) in females (Table 3).

**Table 3:** Intracranial hemorrhage, age, and gender

Gender and age	Hemorrhages	Energy of trauma			
		Low	Medium	High	n/a
Male (*N* hemorrhages)	45	26	9	2	8
Mean age [years (± SD)]	74 (± 12.96)	79 (± 10.16)	69 (± 10.54)	67 (± 18.38)	67 (± 15.53)
Female (*N* hemorrhages)	25	17	1	2	5
Mean age [years (± SD)]	77 (± 19,21)	82 (± 10.7)	45	58,5 (± 30.41)	73 (± 31.56)

Of the 1638 patients, 1469 (89.7%) had no thrombocyte aggregation inhibitor, and 169 (11.3%) had some sort of thrombocyte aggregation inhibitor, where 75 mg ASA accounted for most cases (148/1638). The other 21 patients had treatment with Clopidrogrel 75 mg, Ticagrelor, or a combination of these.

Moreover, 1,505 (91.9%) had no anticoagulation, 119 (7.3%) had warfarin, and 18 (1.1%) had oral anticoagulants or injections of low-molecular-weight heparin with varying dosage.

Of the patients, 23.3% were intoxicated with alcohol, and 25% in total were intoxicated with some kind of drug or a combination of drugs.

Moreover, 92% were assessed using the Reaction Level Scale 1 (RLS1) on their arrival to the emergency department. Of these, 28% had deteriorating mental status (i.e., went from RLS1 to RLS2, etc.) during their emergency-department stay. Observed deterioration of mental status was not associated with higher frequency of intracranial hemorrhage.

Patients without anticoagulants and platelet inhibitors who suffered head trauma had a 3% (40/1351) incidence of intracranial hemorrhage. The hemorrhage frequency in the entire population was 4.3% (70/1638). Among patients on anticoagulants, the frequency of intracranial hemorrhage was 8.6% (10/116), and it was 11.8% (20/169) among those on platelet inhibitors (Table 3).

S100b was measured in 198/1638 cases. Sensitivity was 87.5%, specificity was 46.0%, the negative predictive value was 0.989, and the positive predictive value was 0.064 (Table 4).

**Table 4:** S100b levels and intracranial hemorrhage

s100b Level	Intracranial hemorrhage	
	Yes	No
> 0.1 umol/l	7	102
< 0.1 umol/l	1	87
Not measured	62	1379

## Discussion

This study showed that high age, male gender, low trauma energy, low degree of head injury, and new neurological deficits were correlated with intracranial hemorrhage. These correlations are in accordance with the previous studies and do not reveal any new clinically significant information [1, 14]. To focus on the patient group presenting with the chief complaint ‘head trauma’ and to identify the common traits for this group of patients, all high-energy multitrauma patients were excluded.

Because of the results of the logistic regression analysis, subgroup analysis was performed. This yielded the large group of patients 58 years or younger who did not have any intracranial hemorrhage when trauma energy was low and who did not take anticoagulation and/or antiplatelet therapy. However, because of unreliable patient history with regard to trauma energy in two of the cases, hemorrhages were found also in this age cohort. The in-depth analysis of these two cases revealed that trauma energy level could not be determined.

The validity of this subgroup finding is debatable because of missing data, but it certainly merits further prospective studies. A retrospective analysis of this kind has two main issues of bias to take into account. First, the data are entered into medical records by different physicians who all make individual and slightly different assessments. Second, the medical record data were interpreted by another person and converted to categorical data. We tried to reduce this bias using a rigorous set of rules for how data should be categorized during data collection. We are currently planning a study to evaluate the validity of the present findings, where data will be prospectively recorded and categorized upon collection to minimize bias.

In the Canadian CT Head Rule, medium trauma energy is used as a “rule-in criterion” for head CT, regardless of clinical presentation. However, low trauma energy is not used as a “rule-out criterion.” In the case of low trauma energy, signs and symptoms guide the decision of whether to use CT [1]. In the New Orleans Criteria and Scandinavian Neurotrauma Committee guidelines, trauma energy is not used as a predictor at all [14, 18]. In all three guidelines, level of consciousness is used as a predictor and moderate head injury prompts CT. This is not contradicted by the present study (patients with all degrees of head injury were found both in the 58 years or younger subgroup and in the entire cohort), but it suggests that a higher degree of head injury does not have to be a “rule-in criterion” if other criteria, such as the characteristics of the aforementioned subgroup, are met.

The present study indicates the possibility of ruling out intracranial hemorrhage in some patients based on patient history alone, regardless of clinical status.

The epidemiological analysis shows slightly higher average age compared to the previous studies However, this trend was demonstrated by Roozenbeek et al. in a large meta-analysis [19]. This indicates that the trend has not yet culminated. The distribution between degrees of head injury is largely the same as in studies from the past decade [9, 12, 20]. Because of these findings, which are in line with those of the previous studies, we conclude that the present study has a relevant patient cohort with statistical validity.

The present study indicates a higher frequency of intracranial hemorrhage in patients with antiplatelet medication than in patients with anticoagulants. These results are in accordance with the previous studies by Nishijima et al. [21]. Both our findings and those of Nishijima et al. indicate that guidelines should give as much attention to antiplatelet therapy as to anticoagulation therapy, which is not the case today [1].

The study design, a retrospective review of medical records, has certain limitations. Therefore, one has to be careful not to jump to conclusions based on its results. However, if these findings are reproducible in a prospective study, almost half of the adult patients with traumatic head injury may be safely discharged by the triage nurse based on patient history alone. This would eliminate further medical examination and irradiation and warrants further evaluation in a prospective study, where patient history and trauma energy play a central role in management.

## Conclusion

This retrospective study demonstrates that patients younger than 59 years, with low-energy head trauma who were not on anticoagulants or platelet inhibitors could have been discharged after complete patient history and may not have needed as extensive medical attention as currently recommended and here given. These findings merit further studies.
